# Genome Sequence of the Obligate Gammaproteobacterial Methanotroph *Methylomicrobium album* Strain BG8

**DOI:** 10.1128/genomeA.00170-13

**Published:** 2013-04-11

**Authors:** K. Dimitri Kits, Marina G. Kalyuzhnaya, Martin G. Klotz, Mike S. M. Jetten, Huub J. M. Op den Camp, Stéphane Vuilleumier, Françoise Bringel, Alan A. DiSpirito, J. Colin Murrell, D. Bruce, J.-F. Cheng, A. Copeland, Lynne Goodwin, Loren Hauser, Aurélie Lajus, M. L. Land, A. Lapidus, S. Lucas, Claudine Médigue, S. Pitluck, Tanja Woyke, A. Zeytun, Lisa Y. Stein

**Affiliations:** Department of Biological Sciences, University of Alberta, Edmonton, Alberta, Canadaa;; Department of Microbiology, University of Washington, Seattle, Washington, USAb;; Department of Biology, University of North Carolina, Charlotte, North Carolina, USAc;; Department of Microbiology, IWWR, Radboud University Nijmegen, Nijmegen, the Netherlandsd;; Université de Strasbourg, UMR, CNRS, Strasbourg, Francee;; Department of Biochemistry, Biophysics and Molecular Biology, Ames, Iowa, USAf;; School of Environmental Sciences, University of East Anglia, Norwich, United Kingdomg;; Los Alamos National Lab, Los Alamos, New Mexico, USAh;; U.S. Department of Energy; (DOE), Joint Genome Institute, Walnut Creek, California, USAi;; Oak Ridge National Lab, Biosciences Division, Oak Ridge, Tennessee, USAj;; Laboratoire d’Analyse Bioinformatique en Génomique et Métabolisme, Genoscope-IG-CEA, Evry, Francek

## Abstract

The complete genome sequence of *Methylomicrobium album* strain BG8, a methane-oxidizing gammaproteobacterium isolated from freshwater, is reported. Aside from a conserved inventory of genes for growth on single-carbon compounds, *M. album* BG8 carries a range of gene inventories for additional carbon and nitrogen transformations but no genes for growth on multicarbon substrates or for N fixation.

## GENOME ANNOUNCEMENT

Methanotrophic bacteria are found in diverse environments and utilize methane as their sole source of energy, reductants, and carbon (1). Methanotrophs attenuate the emission of methane, the second most important greenhouse gas (2), and have applications in bioremediation and bioprocessing (3). *Methylomicrobium album* strain BG8 (also known as *Methylobacter albus*, *Methylomonas albus*, and *Methylomonas alba*) is a mesophilic, aerobic gammaproteobacterium isolated from freshwater by Roger Whittenbury et al. (4).

The *M. album* BG8 genome was sequenced, assembled, and annotated by the U.S. Department of Energy Joint Genome Institute (http://www.jgi.doe.gov/sequencing/). Illumina GA II and 454 Titanium standard libraries with paired-end reads were generated, representing 30-fold coverage. Using Newbler v2.3, a chromosomal sequence of 2 contigs and 1 scaffold and a complete plasmid sequence were assembled. Automatic annotation was performed using Prodigal and GenePRIMP (5). The draft genome is 4.49 Mbp, with a mean G+C content of 56.2%. Two copies of the rRNA operon, 42 tRNA genes, and 3,984 predicted protein-coding genes are present. Manual annotation and comparative analysis are under way with assistance from the MicroScope annotation platform at Genoscope (6).

The *M. album* BG8 genome contains one operon (*pmoCAB*) with genes encoding particulate methane monooxygenase and a *pxm* operon (*pxmABC*) with genes encoding a copper membrane monooxygenase of unknown function (7). Genes encoding the enzyme methanol dehydrogenase and accessory proteins (*mxaYDFGIRSACKL-mxaB*) and a Xox-type methanol dehydrogenase (*xoxFJ*) (8) were identified. Genes encoding enzymes for C_1_ metabolism include those for the tetrahydrofolate (H_4_-folate) and tetrahydromethanopterin (H_4_MPT)-linked C_1_ transfer pathways, glutathione-dependent formaldehyde dehydrogenases (GD-FALDH) and accessory functions, and a NAD-dependent formate dehydrogenase (encoded by *fdh5A*). A membrane-bound formate dehydrogenase (encoded by *fdh3DABC*) is typically absent in strict methanotrophs, but it is expressed in “*Candidatus* Methylacidiphilum fumariolicum” SolV of the phylum *Verrucomicrobia*. Also similar to “*Ca*. Methylacidiphilum fumariolicum” SolV, *M. album* BG8 was found to have a complete set of genes for glycogen biosynthesis (9). Genes encoding the complete ribulose monophosphate (RuMP) pathway for formaldehyde assimilation, a complete tricarboxylic acid (TCA) cycle, the pentose phosphate pathway, and the Embden-Meyerhof-Parnas pathway were identified. A complement of genes for a complete serine cycle, with the exception of phosphoenolpyruvate carboxylase, was identified; key genes for the Calvin-Benson-Bassham cycle were absent.

Genes encoding enzymes for nitrogen metabolism were identified, including those for direct ammonium uptake (*amtB*), nitrate transport (*narK*), nitrate or nitrite reduction (*nasCA, nasB*, and *nirBD*), and glutamine synthetase/glutamate synthase (*glnA, gltB*) and alanine dehydrogenase (*ald*) for ammonium assimilation. Tandem genes encoding proteins implicated in the oxidation of hydroxylamine to nitrite (*haoAB*) were identified, which likely facilitate the detoxification of hydroxylamine produced from the oxidation of ammonia by membrane-bound methane monooxygenase (pMMO) (10). Genes encoding a cytochrome *cd*_1_ nitrite reductase (*nirS*) and accessory functions, as well as nitric oxide redutase (*norCB*), were found, perhaps explaining the capacity of this strain to tolerate high nitrite concentrations (11). The *nirS* and *norCB* genes in this strain share high sequence identities with homologues in other methanotrophs (12, 13). Analysis of the *M. album* BG8 genome sequence enables further understanding of single-carbon metabolism and the environmental adaptation strategies of methanotrophs.

### Nucleotide sequence accession numbers. 

The genome sequences of the chromosome and plasmid of *M. album* BG8 have been deposited in GenBank under accession no. CM001475 and CM001476, respectively.
